# Heterologous synthesis of chlorophyll *b* in *Nannochloropsis salina* enhances growth and lipid production by increasing photosynthetic efficiency

**DOI:** 10.1186/s13068-019-1462-3

**Published:** 2019-05-14

**Authors:** Hyun Gi Koh, Nam Kyu Kang, Seungjib Jeon, Sung-Eun Shin, Byeong-ryool Jeong, Yong Keun Chang

**Affiliations:** 1 Advanced Biomass R&D Center, 291 Daehak-ro, Yuseong-gu, Daejeon, 34141 Republic of Korea; 2 Department of Chemical and Biomolecular Engineering, KAIST, 291 Daehak-ro, Yuseong-gu, Daejeon, 34141 Republic of Korea; 3 Present Address: Carl R. Woese Institute for Genomic Biology, University of Illinois at Urbana-Champaign, Urbana, IL USA; 4 Present Address: LG Chem, 188 Munji-ro, Yuseong-gu, Daejeon, 34122 Republic of Korea

**Keywords:** Microalgae, *Nannochloropsis salina*, Chlorophyll *b*, Chlorophyllide *a* oxygenase (CAO), Biofuels, Photosynthesis

## Abstract

**Background:**

Chlorophylls play important roles in photosynthesis, and thus are critical for growth and related metabolic pathways in photosynthetic organisms. They are particularly important in microalgae, emerging as the next generation feedstock for biomass and biofuels. *Nannochloropsis* are industrial microalgae for these purposes, but are peculiar in that they lack accessory chlorophylls. In addition, the localization of heterologous proteins to the chloroplast of *Nannochloropsis* has not been fully studied, due to the secondary plastid surrounded by four membranes. This study addressed questions of correct localization and functional benefits of heterologous expression of chlorophyllide *a* oxygenase from *Chlamydomonas* (CrCAO) in *Nannochloropsis*.

**Results:**

We cloned *CrCAO* from *Chlamydomonas*, which catalyzes oxidation of Chl*a* producing Chl*b*, and overexpressed it in *N. salina* to reveal effects of the heterologous Chl*b* for photosynthesis, growth, and lipid production. For correct localization of CrCAO into the secondary plastid in *N. salina*, we added the signal-recognition sequence and the transit peptide (cloned from an endogenous chloroplast-localized protein) to the N terminus of CrCAO. We obtained two transformants that expressed CrCAO and produced Chl*b*. They showed improved growth under medium light (90 μmol/m^2^/s) conditions, and their photosynthetic efficiency was increased compared to WT. They also showed increased expression of certain photosynthetic proteins, accompanied by an increased maximum electron-transfer rate up to 15.8% and quantum yields up to 17%, likely supporting the faster growth. This improved growth resulted in increased biomass production, and more importantly lipid productivity particularly with medium light.

**Conclusions:**

We demonstrated beneficial effects of heterologous expression of CrCAO in Chl*b*-less organism *N. salina*, where the newly produced Chl*b* enhanced photosynthesis and growth. Accordingly, transformants showed improved production of biomass and lipids, important traits of microalgae from the industrial perspectives. Our transformants are the first *Nannochloropsis* cells that produced Chl*b* in the whole evolutionary path. We also succeeded in delivering a heterologous protein into the secondary plastid for the first time in *Nannochloropsis*. Taken together, our data showed that manipulation of photosynthetic pigments, including Chl*b*, can be employed in genetic improvements of microalgae for production of biofuels and other biomaterials.

**Electronic supplementary material:**

The online version of this article (10.1186/s13068-019-1462-3) contains supplementary material, which is available to authorized users.

## Background

Chlorophylls are photosynthetic pigments that play important roles in oxygenic photosynthesis in cyanobacteria, algae, and plants. Photosynthesis harnesses the sun’s energy to produce oxygen and to fix carbons, which provide basic necessities for all life on Earth. In particular, photosynthesis in microalgae is emerging as valuable sources for the next-generation biomass and biofuels. Improving microalgal photosynthesis is thus critical for successful production of sustainable biomaterials, and understanding functions of chlorophylls is one of the keys to successful employment of microalgae. In fact, engineering of photosynthesis is being actively pursued to improve production of biomass in crops and microalgae [1].

Chlorophylls (Chls) are magnesium-tetrapyrrole molecules, and have functions in harvesting light energy in the light-harvesting complexes (LHCs) and in driving charge-separation reactions in the reaction centers [2]. Chlorophylls are evolutionarily conserved, and are used as taxonomic keys for photosynthetic organisms [3]. Chlorophyll *a* (Chl*a*) is the universal photosynthetic pigment that is present in most oxygenic photosynthetic organisms, and performs all of the above functions [4]. Chl*b* and other accessory pigments are mainly involved in light harvesting in LHCs, and are expected to improve light-harvesting efficiency [2, 5, 6]. Chl*c* is another accessory pigment involved in light harvesting, and is found in heterokont algae descended from secondary endosymbiosis [7]. Heterokonts are polyphyletic and are represented by diatoms and *Nannochloropsis*; however, *Nannochloropsis* have lost Chl*c*, and carotenoids have taken over the functions of accessory pigments [8, 9].

**Fig. 1:**
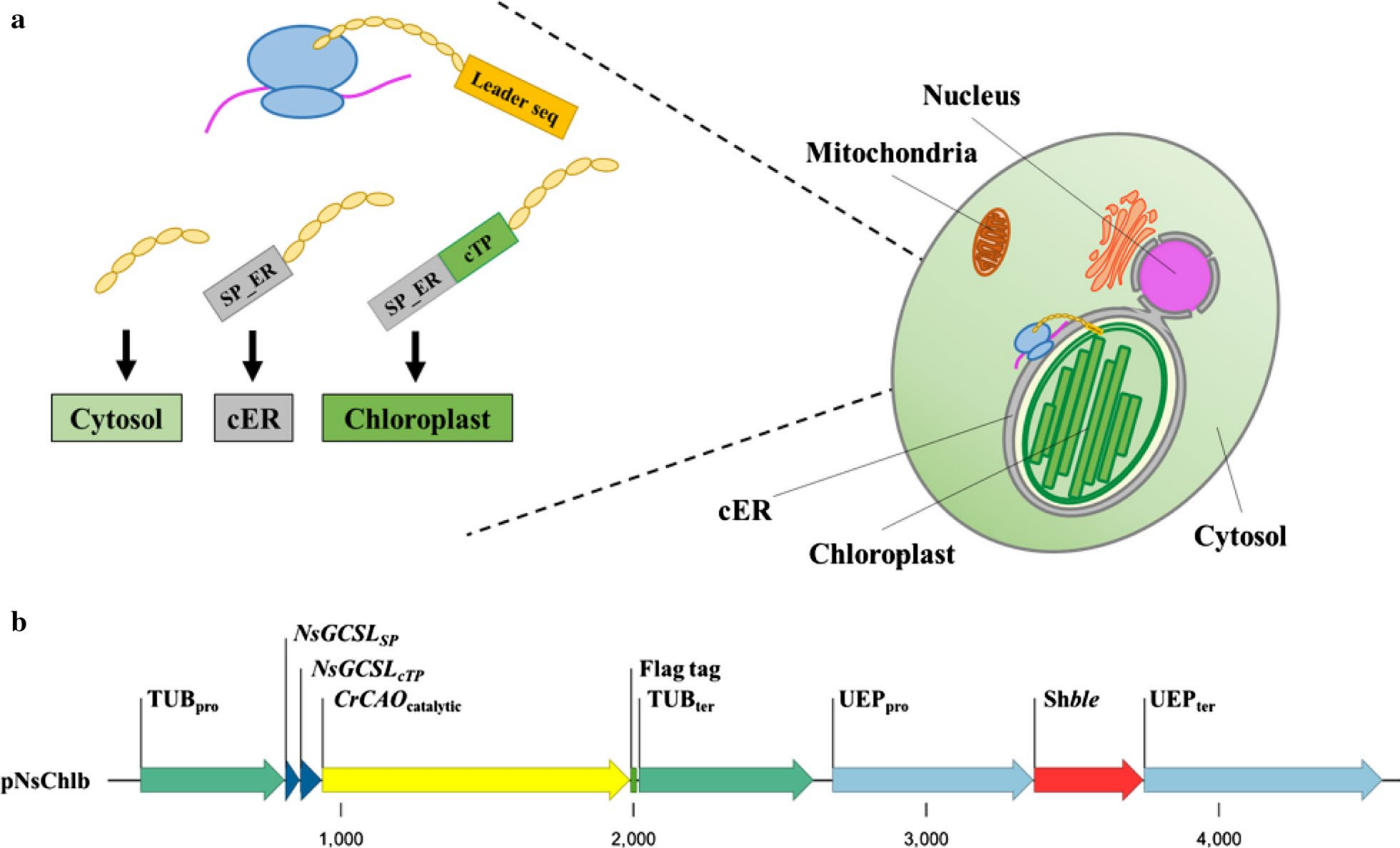
Schematic drawings of chloroplast and cER localization in *Nannochloropsis*, and vector construction. **a** Simplified cellular structure of *Nannochloropsis* including organelles, in particular, the chloroplast and the surrounding cER. Therefore, chloroplast localization of proteins requires signals for the signal peptide for cER (SP-ER) and the chloroplast transit peptide (cTP). **b** Construction of the pNsChlb vector for expression of the catalytic domain of CrCAO cloned from *Chlamydomonas reinhardtii*. For proper localization of CAO to the chloroplast, signal peptide and cTP from the *GCSL* gene from *N. salina* were added to CrCAO. The recombinant CrCAO was tagged with the Flag tag, and the vector contained the Sh*ble* marker

Lack of accessory chlorophylls in *Nannochloropsis* is interesting in that they have been known for robust growth and lipid production. In fact, *Nannochloropsis* are considered model microalgae with remarkable potential for industrial production of biomass and lipids [10–13]. However, their photosynthetic efficiency has been in question due to the lack of pyrenoids that serve as a carbon concentration mechanism in many microalgae, even though alternative mechanism(s) may exist [12, 14, 15]. Accessory chlorophylls are known to improve photosynthesis in part by extending the light absorption spectrum and antennal assembly, which can also affect growth and hormonal responses [2, 5]. It would be interesting to test if an accessory chlorophyll would be beneficial for photosynthesis in *Nannochloropsis*. Currently, genetic information about Chl*c* biosynthesis is unknown [7, 16]; however, Chl*b* is produced simply by successive hydroxylations of chlorophyllide *a* or Chl*a*, catalyzed by a single enzyme called chlorophyllide *a* oxygenase (CAO) in *Chlamydomonas* [2, 17, 18], opening the possibility to produce Chl*b* in *Nannochloropsis*. Considering the reports of remarkable increase in starch contents and growth rate after overproduction of Chl*b* [19] in *Nicotiana tabacum*, there is a possibility of positive effect by heterologous synthesis of Chl*b* in *N. salina* as well. We expressed the catalytic domain of CAO from *Chlamydomonas* in *N. salina* CCMP 1776 to determine if Chl*b* produced by the heterologous CAO had beneficial effects on growth and photosynthesis. CAOs contain three domains, called A, B, and C domains, in *Chlamydomonas* and plants, among which the C domain is catalytic, while the A and B domains are regulatory [17, 20]. Interestingly, the A and B domains are missing in other microalgae and cyanobacterium *Prochlorothrix hollandica*, and the C domain is sufficient for biochemical functions of CAO [17, 21, 22]. For proper delivery of CAO into the chloroplast endoplasmic reticulum (cER) and the chloroplast in *Nannochloropsis*, we added signal and transit peptide sequences from a chloroplast-localized protein Glycine Cleavage System L protein (GCSL, encoding dihydrolipoamide dehydrogenase) in *Nannochloropsis* at the N terminus of CAO. cER originated from the secondary symbiosis of a red algal ancestor, which is a characteristic of heterokonts [14, 23], and localization requires the signal peptide prior to the chloroplast localization [24, 25], as summarized in Fig. 1a.

Expression of the recombinant CAO in *Nannochloropsis* resulted in the production of Chl*b* with improved photosynthesis and growth, and most importantly, with the increased lipid productivity. Improved photosynthesis by CAO is consistent with the previous findings that Chl*b* is also involved in antennal assembly and optogenetic signaling in plants and *Chlamydomonas* [5, 6, 26]. We also show that the improvements were further enhanced under low light conditions, suggesting that production of Chl*b* contributed to more efficient light harvesting. To our best knowledge, this is the first study to produce the accessory Chl*b* in heterokonts and to express heterologous protein in cER in *Nannochloropsis* by adding the signal and transit peptide sequences. Conclusively, this report provides an excellent proof of concept that simple introduction of CAO into industrial heterokonts could improve production of biomass and lipids.

## Results

### Construction of CAO expression vector (pNsChlb) and transformation into *N. salina* CCMP 1776

We cloned the catalytic domain of the *CAO* gene from *Chlamydomonas reinhardtii* CC-124 (*CrCAO*; GenBank accession: XM_001690123.1) via RT-PCR and inserted it downstream of the *TUB* promoter, resulting in the pNsChlb vector (Fig. 1b). To ensure proper localization to the chloroplast, we fused the signal peptide and chloroplast transit peptide (cTP) from a chloroplast-localized protein GCSL (Additional file 1: Figure S1) at the N terminus of CrCAO. The GCSL sequence was initially found in the genome of *N. gaditana* (Genbank accession: Naga_100001g181) via the Nannochloropsis Genome Portal (http://www.nannochloropsis.org/) [27], and was amplified from the cDNA of *N. salina* CCMP1776 using primers GC1 and GC2 (Additional file 2: Table S1). The signal peptide and transit peptide of GCSL were confirmed by SignalP 4.1 [28], ChloroP [29], and HECTAR [30] (Additional file 3: Figure S2). The chimeric CrCAO was tagged with ‘Flag’ at the C terminus for western detection, and the vector contained the Sh*ble* marker gene that allowed selection of transformants with an antibiotic Zeocin.

**Fig. 2:**
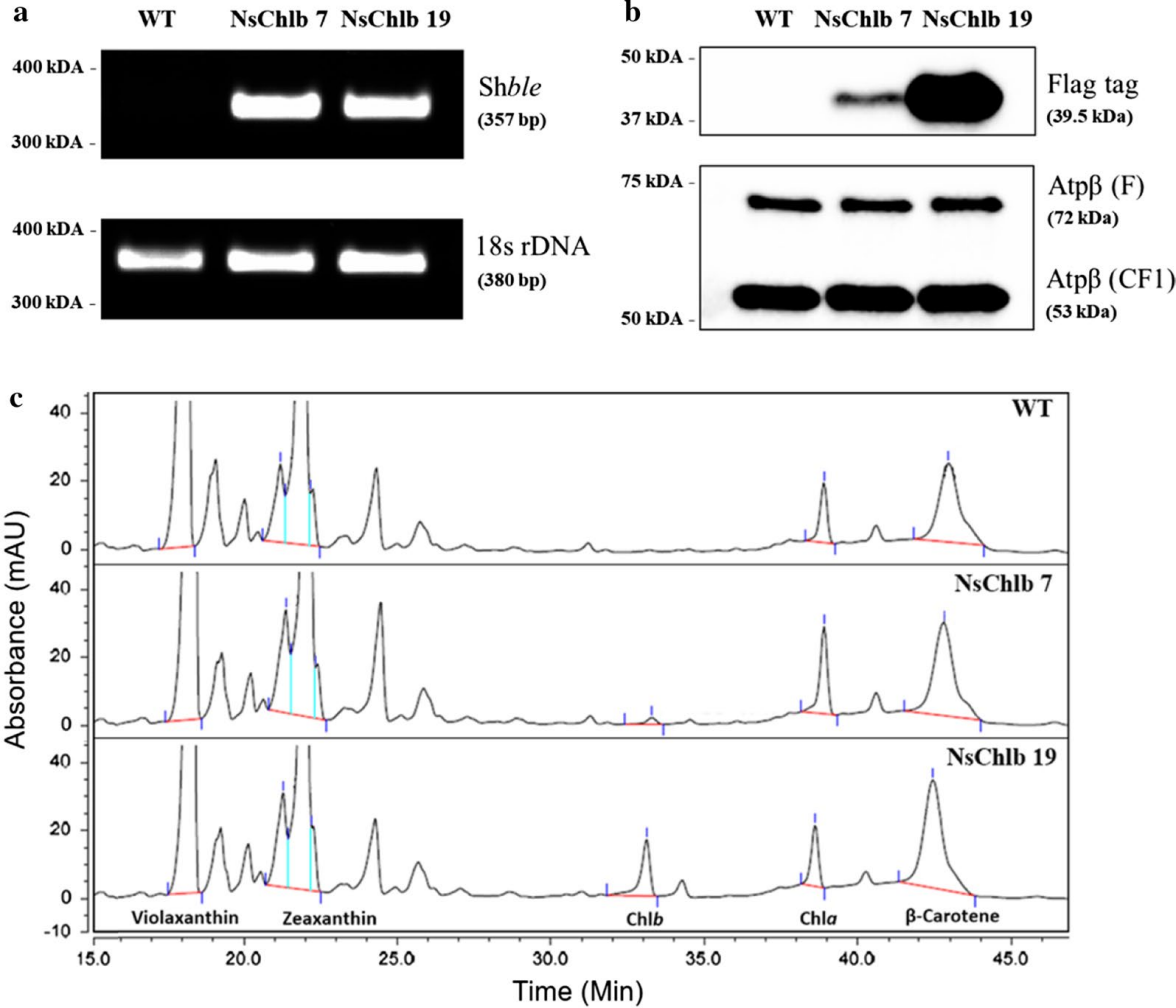
Characterization of transformants of CrCAO. **a** PCR detection of the inserted plasmid (pNsChlb) by amplifying the 375 bp Sh*ble* sequence only in transformants NsChlb7 and NsChlb19. The 380 bp product of 18S rDNA was used as a loading control and was detected in all samples. **b** Western blotting for expression of the FLAG-tagged CrCAO protein from transformants. The Flag tag was detected only in transformants, while the β subunit of ATP synthase (Atpβ) appeared as the F-type H-ATPase β subunit (72.6 kDa) and the CF1 β subunit (53.13 kDa) in WT and transformants. **c** Detection of chlorophylls and other photosynthetic pigments via HPLC at 460 nm absorbance. Peaks for chlorophyll *a* and a few carotenoids including β-carotene and violaxanthin were shown. Emergence of chlorophyll *b* was detected only in the transformants

Transformants were selected on F2 N agar plates containing 2.5 μg/mL of Zeocin after electroporation, and we confirmed the presence of the vector sequence via genomic PCR (Fig. 2a) and the expression of CrCAO::Flag via western blot for Flag (Fig. 2b). Integration of the vector was confirmed by RESDA-PCR (restriction enzyme site-directed amplification-polymerase chain reaction), revealing integration at the UTR of a hypothetical gene (homolog of Naga_101464g1.1 in *N. gaditana* B-31) in NsChlb7 and in an intergenic region near a gene (homologous to Naga_100033g27 in *N. gaditana* B-31 encoding phosphatase 2C) in NsChlb19, which do not appear to affect phenotypes of transformants (Additional file 4: Figure S3). The chimeric CrCAO was detectable only in transformants NsChlb 7 and NsChlb 19 at 39.5 kDa. It should be noted that this size is expected for the processed protein in the chloroplast, instead of the full length (43.88 kDa), suggesting successful removal of the signal and transit peptide sequences during translocation to the chloroplast. Actual localization of the produced CrCAO proteins are further confirmed by immuno-gold labeling technique with transmission electron microscopy (Additional file 5: Figure S4).

Production of Chl*b* was analyzed with high-performance liquid chromatography (HPLC), as shown in Fig. 2c. Chl*a* and other photosynthetic pigments were well represented in all samples, while the peak for Chl*b* was only detectable in transformants. Interestingly, NsChlb 19 showed more accumulation of Chl*b* than NsChlb 7, consistent with higher expression of CrCAO protein, as shown Fig. 2b. These two transformants, NsChlb 7 and NsChlb 19, were used for further analyses.

### Effects of CrCAO on growth under different light intensities

**Fig. 3:**
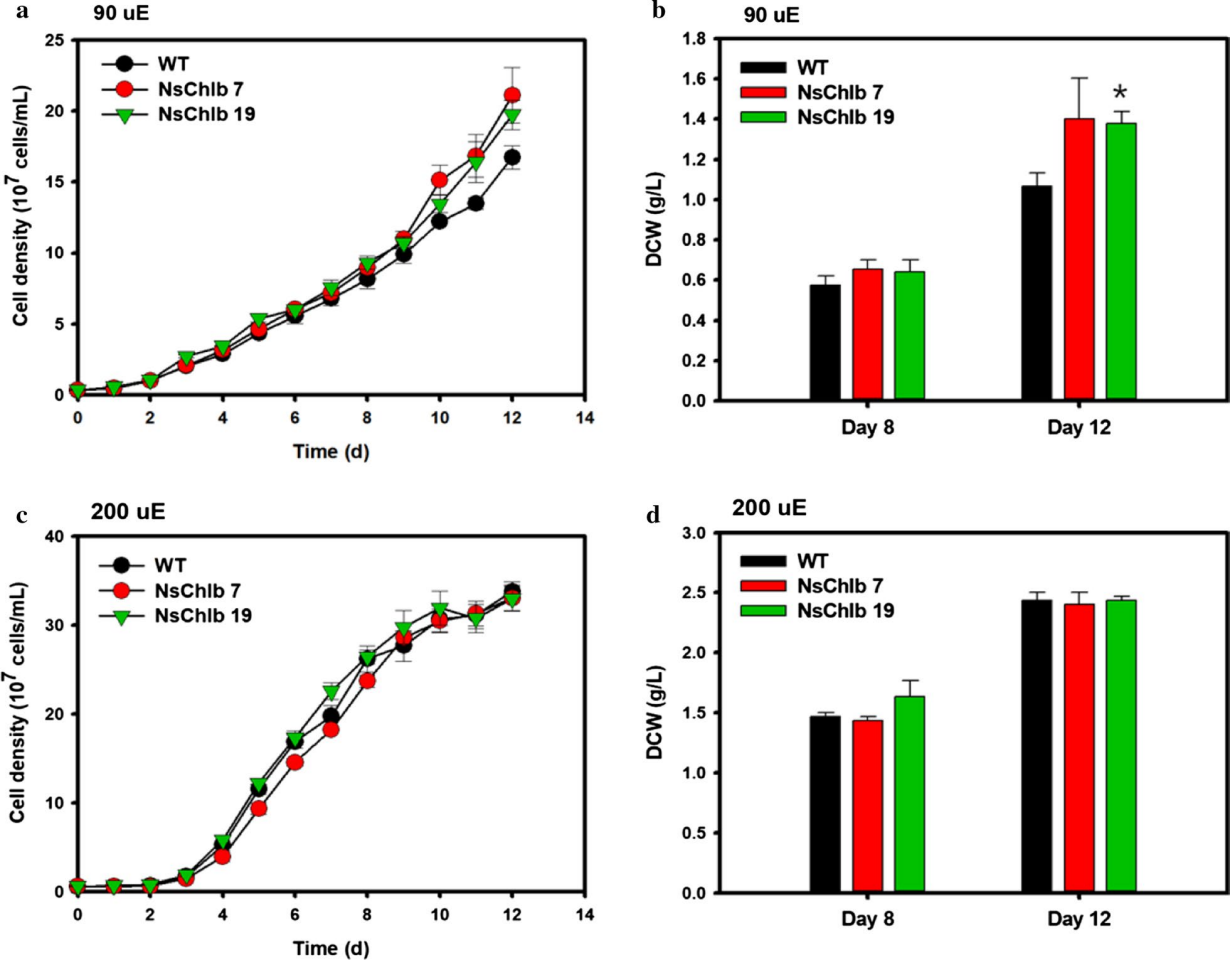
Growth analyses of Chl*b* synthesizing transformants under different light intensities of medium light (ML: 90 μmol photons/m^2^/s) and high light (HL: 200 μmol photons/m^2^/s). The cell density (**a**, **c**) and dry cell weight (DCW: **b**, **d**) were analyzed under ML (**a**, **b**) and HL (**c**, **d**). Data points represent means and standard errors (*n* = 3). Asterisks indicate the significant differences between WT and each transformants (WT vs. NsChlb7, WT vs. NsChlb19) determined by Student’s *t* test (*P < 0.05, **P < 0.01, ***P < 0.001)

We analyzed the growths of WT, NsChlb7, and NsChlb 19 under high (HL: 200 μmol photons/m^2^/s) and medium light intensities (ML: 90 μmol photons/m^2^/s) to reveal beneficial effects of Chl*b* on growth under different light conditions (Fig. 3). Growths estimated by cell density (Fig. 3a) and dried cell weight (DCW; Fig. 3b) showed improvements in transformants under medium light in NsChlb19, particularly during the later phase of growth (days 10–12). The cell number on day 12 was 18–26% higher in transformants compared to that of the WT with ML, with a concomitant increase in DCW of 29–31%, where NsChlb7 showed similar improvements but no significance. However, there were no significant differences in growth for the transformants under HL (Fig. 3c, d), suggesting that the extra Chl*b* contributed to growth under ML.

### Photosynthetic parameters and pigments

**Fig. 4:**
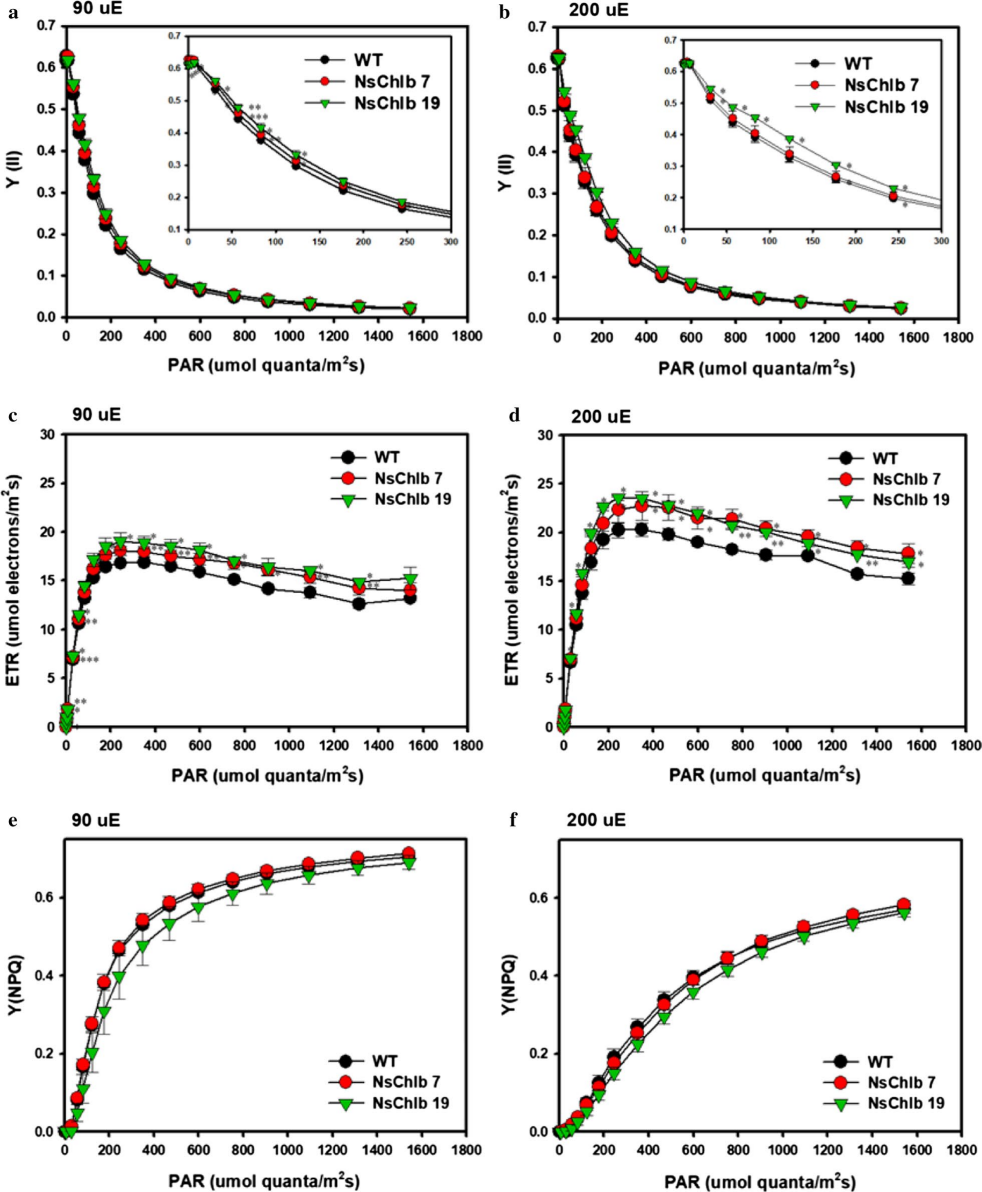
Comparison of photosynthetic parameters in WT and transformants employing Pulse-Amplitude-Modulation (PAM) fluorometry using cells grown under ML (90 uE) and HL (200 uE). The quantum yields at the increasing light intensity of strains cultivated under ML (**a**) and HL (**b**). Insets show close-up values of measurements with light intensity of < 300 μE. The electron-transfer rates (ETRs) with the increasing light intensity under ML (**c**) and HL (**d**). Changes in non-photo chemical quenching (NPQ) yield with the increasing light intensity under ML (**e**) and HL (**f**). Data points represent means and standard errors (*n* = 3). Asterisks indicate the significant differences between WT and each transformants (WT vs. NsChlb7, WT vs. NsChlb19) determined by Student’s *t* test (*P < 0.05, **P < 0.01, ***P < 0.001)

We further analyzed photosynthetic efficiency and pigments that may be contributed by the novel production of Chl*b*, as summarized in Fig. 4 and Table 1. Cells were cultured with ML and HL, and were subjected to photometric measurement of photosynthesis using Multi-color-PAM. The maximum quantum yield of photosystem II (Fv/Fm) was in the range of 0.62 ± 0.02 in all strains and did not show differences between WT and transformants (Fig. 4a, b), in agreement with the relatively constant maximum quantum yield in other photosynthetic organisms under no environmental stresses [31]. However, transformants showed moderate but significant improvements in quantum yield measured with higher light intensities, particularly in NsChlb 19, which expressed more CAO proteins (inlets in Fig. 4a, b). Transformants also showed improved electron transfer rates compared to WT (Fig. 4c, d). We also measured the yield of non-photochemical quenching (NPQ) for heat dissipation of light energy received through the light-harvesting complex (LHC), but did not find any significant differences in samples (Fig. 4e, f). Overall, transformants showed improved quantum yield and electron transport, and a moderate reduction in NPQ, which could contribute to improved growth under ML.

**Table 1:** Photosynthetic pigments analyzed by HPLC

(Scale: μg/mg)	Pigments	ML (90 μE)			HL (200 μE)		
		WT	NsChlb 7	NsChlb 19	WT	NsChlb 7	NsChlb 19
Day 8	Chl*a*	45.36 ± 3.13	57.61 ± 1.69*	50.09 ± 1.09	43.92 ± 3.42	50.70 ± 2.30	39.48 ± 2.44
	Chl*b*	n.d.	0.28 ± 0.05	1.51 ± 0.13	n.d.	0.18 + 0.01	0.53 + 0.07
	Violaxanthin	3.88 ± 0.06	4.48 ± 0.09	4.04 ± 0.17	4.10 ± 0.29	5.06 ± 0.26*	3.90 ± 0.21
	β-carotene	4.42 ± 0.45	4.07 ± 0.83	4.58 ± 0.93	1.08 ± 0.42	2.36 ± 0.06**	1.85 ± 0.07*
	Zeaxanthin	0.09 ± 0.01	0.12 ± 0.01	0.16 ± 0.01	0.10 ± 0.01	0.11 ± 0.01	0.10 ± 0.01
Day 12	Chl*a*	72.52 ± 1.90	75.86 ± 8.84	70.55 ± 4.43	23.25 ± 1.30	25.86 ± 1.16	20.23 ± 1.30
	Chl*b*	n.d.	0.15 ± 0.07	0.97 ± 0.40	n.d.	0.05 ± 0.02	0.13 ± 0.05
	Violaxanthin	7.37 ± 0.08	7.52 ± 1.22	7.42 ± 0.29	1.81 ± 0.19	2.10 ± 0.23	1.70 ± 0.17
	β-carotene	5.68 ± 0.09	5.84 ± 0.55	5.49 ± 0.19	1.39 ± 0.10	1.67 ± 0.17	1.34 ± 0.02
	Zeaxanthin	0.12 ± 0.01	0.11 ± 0.01	0.09 ± 0.20	0.17 ± 0.01	0.20 ± 0.03	0.13

The data represent the average value with ranges of standard error (*n* = 3). Significant differences between WT and each transformants (WT vs. NsChlb7, WT vs. NsChlb19) are determined by Student’s *t* test and are indicated by asterisks (*P < 0.05, **P < 0.01, ***P < 0.001)

We also measured chlorophyll and carotenoid contents in greater detail, after cultivation for 8 and 12 days (Table 1). Likely through the catalytic activity of heterologous CrCAO, Chl*b* was detectable only in transformants NsChlb 7 and NsChlb 19, which was also shown in the HPLC profile (Fig. 2c). As described earlier, NsChlb 19 expressed more CrCAO and produced more Chlb. Chl*a* showed a tendency of increasing content on day 8 under ML, especially in NsChlb7 strain. However, no significant changes were observed in other conditions in both transformants. Chl*a* is converted to Chl*b*; however, we did not observe decreased Chl*a* content in transformants in any cases, likely because only a small fraction of Chl*a* was converted to Chl*b*. Overall, chlorophyll contents decreased under HL compared to ML, probably due to antennal reduction under HL [32]. In addition to chlorophylls, we also measured carotenoids including Violaxanthin, β-carotene, and zeaxanthin. Transformants did not show substantial and/or consistent changes in carotenoid content compared to WT. Taken together, the results indicate that chlorophylls (particularly Chl*b*) may have contributed to the improved photosynthesis in CrCAO transformants.

### Quantitation of antenna and other photosynthetic proteins

**Fig. 5:**
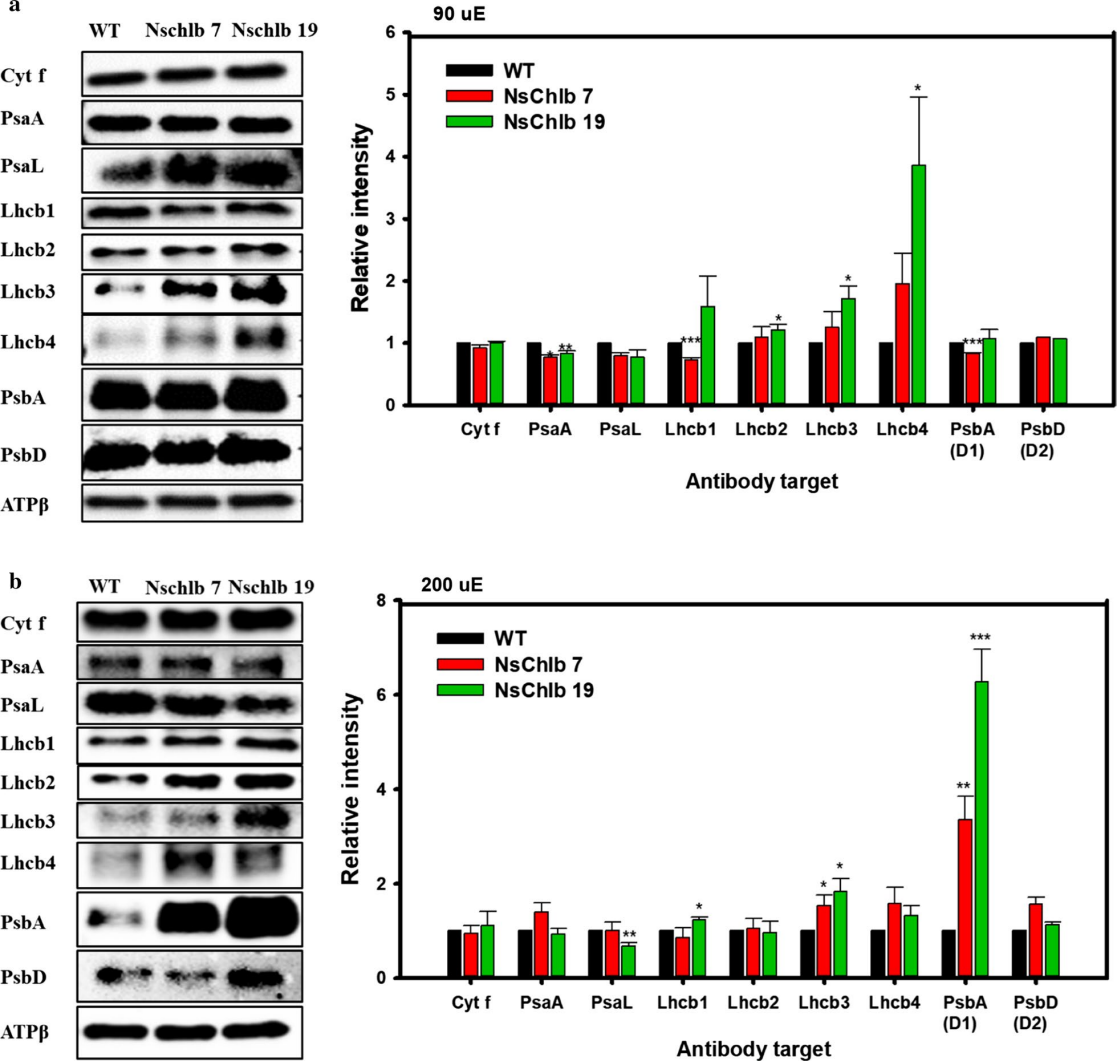
Quantitation of photosynthetic proteins via western blotting and estimation of band intensities. Cells were grown under ML (**a**) or HL (**b**), and 1.5 × 10^8^ cells were harvested on day 8 for western blotting. Band intensities for individual proteins were normalized with that of ATPβ (using the Image Lab software), and relative intensities to WT are presented on the right panel. Each data point represents the average of three independent replicates and error bars are standard errors. Asterisks indicate the significant differences between WT and each transformants (WT vs. NsChlb7, WT vs. NsChlb19) determined by Student’s *t* test (*P < 0.05. **P < 0.01, ***P < 0.001)

For further understanding of the photosynthetic improvements in Chl*b*-producing transformants, we quantitated antenna and other photosynthetic proteins via western blot followed by quantitation of band intensity using Chemi-Doc (Fig. 5). The ATPβ subunit was used as a loading control to normalize the intensity of blotted bands. We did not find any difference in cytochrome f in transformants compared to WT, but found a slight decrease in photosystem I (PSI) components, even though no proteins showed significant differences. However, we observed general trends of increasing LHCII antennal components (Lhcb1-like, Lhcb2-like, Lhcb3-like, and Lhcb4-like proteins), except for Lhcb1 in NsChlb 7 under ML, which might have contributed to increased photosynthesis. For the analysis of the LHCII antennal components, we used each antibody targeting Lhcb1 ~ 4 proteins (Agrisera, USA) in *C. reinhardtii*, as antibodies targeting LHCII proteins in heterokonts are not widely available. Interestingly, Lhcb4-like protein was greatly increased in transformants under ML, albeit with large error bars, which may reflect its accumulation pattern depending on light intensity and/or chlorophyll content in microalgae and plants [5, 33, 34]. PSII core proteins (D1 and D2) were reduced or did not change in transformants under ML, but increased under HL, which may be correlated with stabilizing effect of Chl*b* under light intensive conditions [35]. Overall, we showed that Chl*b* production was associated with enhanced accumulation of LHCII and PSII core proteins, which may contribute to enhanced photosynthesis in CrCAO transformants.

### Assessment of lipid production in CrCAO transformants

**Fig. 6:**
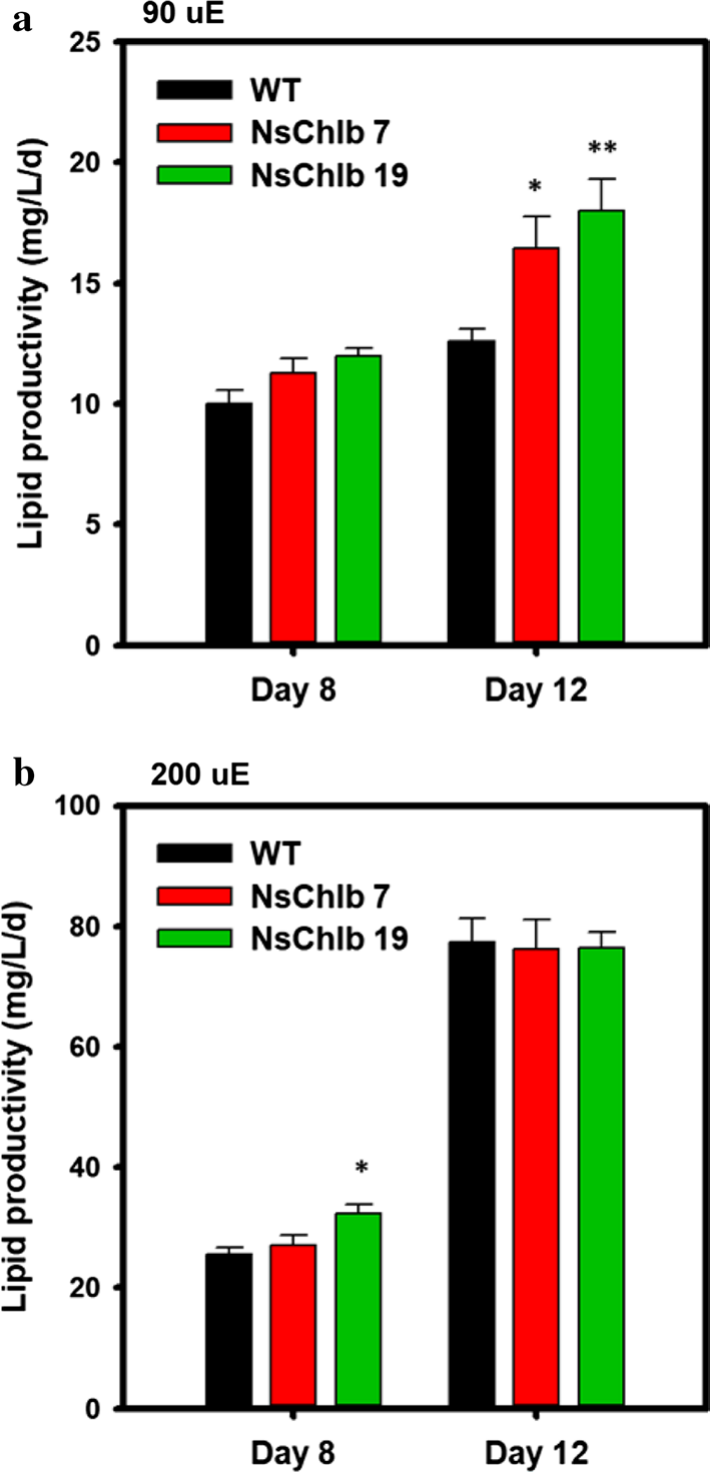
Total lipid productivities of WT and NsChlb transformants. The total lipid productivity was calculated from the biomass productivity and lipid contents divided by the cultivation period in days. Cells were cultivated under ML (90 μmol photons/m^2^/s) (**a**) and HL (200 μmol photons/m^2^/s) (**b**) at 25 °C, 120 rpm and supplying 0.5 vvm of 2% CO_2_. Asterisks indicate the significant differences between WT and each transformants (WT vs. NsChlb7, WT vs. NsChlb19) determined by Student’s *t* test (*P < 0.05, **P < 0.01, ***P < 0.001, *n* = 3)

Lipid production in CrCAO transformants was also analyzed, since they showed improvements in photosynthetic efficiency and growth. We used high performance liquid chromatography (HPLC) to quantitate different types of lipids under ML and HL conditions, and showed lipid contents (Table 2) and lipid productivities (Fig. 6). Overall, TAG contents were not changed substantially, although NsChlb 7 showed a slight but significant decrease in TAG content under ML on day 12. Under HL, transformants showed a tendency of increased TAG content on day 8, which may be correlated to their increased photosynthesis (Fig. 4b, d) without change in growth (Fig. 3c, d). MAG was not detectable on day 8 with ML, but accumulated on day 12, while it was detectable under HL. We did not found any significant changes in MAG content in any samples. Galactolipids, including monogalactosyldiacylglycerol (MGDG) and digalactosyldiacylglycerol (DGDG), which are components of the thylakoid membrane in chloroplasts, were produced more under ML than HL, and more on day 8 than day 12. This pattern may be related to a reduced photosynthetic apparatus under HL or stress conditions, as reported in plants and microalgae [36, 37]. CrCAO transformants showed significantly increased polar lipid content only under ML on day 12, which may be related to their significant improvement in growth under these conditions (Fig. 3a, b).

**Table 2:** Lipid contents of WT and NsChlb transformants grown under different light conditions measured by HPLC

	Strain	TAG	MAG	MGDG	DGDG	Total
ML (90 μE)						
Day 8	WT	3.90 ± 0.14	n.d.	7.77 ± 0.13	2.33 ± 0.05	14.00 ± 0.13
	NsChlb 7	3.57 ± 0.27	n.d.	7.64 ± 0.55	2.56 ± 0.09 *	13.77 ± 0.54
	NsChlb 19	4.93 ± 0.52	n.d.	7.40 ± 0.21	2.79 ± 0.19 *	15.12 ± 0.80
Day 12	WT	3.03 ± 0.08	2.07 ± 0.02	5.05 ± 0.32	2.28 ± 0.02	14.21 ± 0.31
	NsChlb 7	2.76 ± 0.06*	2.01 ± 0.02	6.30 ± 0.29*	2.47 ± 0.03**	14.37 ± 0.95
	NsChlb 19	3.06 ± 0.27	2.01 ± 0.01	6.12 ± 0.19*	2.49 ± 0.05**	15.67 ± 0.69
HL (200 μE)						
Day 8	WT	4.58 ± 0.62	2.89 ± 0.03	3.71 ± 0.06	2.55 ± 0.02	14.01 ± 0.81
	NsChlb 7	5.45 ± 0.56	3.17 ± 0.13	3.62 ± 0.02	2.59 ± 0.06	15.10 ± 0.70
	NsChlb 19	6.98 ± 0.81*	2.84 ± 0.02	3.44 ± 0.13	2.60 ± 0.10	15.94 ± 0.53
Day 12	WT	32.32 ± 1.41	2.51 ± 0.02	2.04 ± 0.05	1.80 ± 0.04	38.67 ± 1.33
	NsChlb 7	31.68 ± 2.14	2.52 ± 0.07	2.06 ± 0.10	1.82 ± 0.07	38.08 ± 1.92
	NsChlb 19	31.43 ± 1.77	2.43 ± 0.03	2.04 ± 0.04	1.78 ± 0.02	37.67 ± 1.71

The data represent the average value with ranges of standard error (*n* = 3). Asterisks indicates the significant differences between WT and each transformants (WT vs. NsChlb7, WT vs. NsChlb19) determined by Student’s *t* test (*P < 0.05, **P < 0.01, ***P < 0.001)

Overall, total lipid contents were similar on day 8 under ML and HL, but increased noticeably on day 12 under HL compared to ML (Table 2). CrCAO transformants did not show any significant changes in total lipid contents under any conditions. However, they showed a significant increase in biomass under ML on day 12 (Fig. 3b), and we estimated the lipid productivity as shown in Fig. 6. As expected, both transformants showed significantly increased lipid productivities on day 12 with ML (Fig. 6a). Under HL, transformants remained the same as WT, although NsChlb 19 showed a moderate but significant increase in lipid productivity on day 8 (Fig. 6b).

## Discussion

*Nannochloropsis* are one of the most important industrial microalgae of which genomic and transcriptomic resources are available [12, 27], and have been subjected to intense strain developments via transcription factor engineering [38–40] and metabolic engineering [41, 42]. Overall, they show robust growth and high lipid contents [43, 44]; however, their photosynthetic efficiency has been questioned due to the lack of pyrenoids and leaky carbon concentrating mechanisms [14, 15, 45]. They are also peculiar in that they lack accessory chlorophylls (Chl*b* or Chl*c*), which is rare in photosynthetic eukaryotes [8, 17, 46]. This opens a unique opportunity to test whether Chl*b* would be beneficial for photosynthesis and growth, and further for lipid production in *Nannochloropsis*.

We found that production of Chl*b* by introducing heterologous CrCAO indeed improved photosynthesis and growth, particularly under ML conditions. These improvements are expected to be caused by the effect of CrCAO, as integration sites of transformants were not found in the coding sequences, revealed by RESDA-PCR (Additional file 4: Figure S3). During the process of introducing heterologous proteins into the chloroplast of *Nannochloropsis*, we learned that the transit peptide was not sufficient for chloroplast localization, because the chloroplast is surrounded by another pair of membranes (forming cER) in heterokonts including *Nannochloropsis* and diatoms, originating from the secondary endosymbiosis of a red algal ancestor [15, 21–23]. In our earlier attempt to express CrCAO without any modification, we failed to obtain any transformants that produced Chl*b*. However, we successfully introduced CrCAO into the chloroplast by fusing a signal-recognition sequence and transit peptide from a chloroplast-localized protein called GCSL, resulting in the production of active Chl*b* in *Nannochloropsis*. The production of Chl*b* and the delivery of a heterologous protein into the chloroplast have not been reported in *Nannochloropsis* except for the cases of marker proteins such as GFP [47].

CrCAO transformants showed improved photosynthetic efficiencies and changed amounts of antennal and photosystem proteins, consistent with functions of Chl*b* and CAO in other photosynthetic organisms. Even though the amount of Chl*b* synthesized in NsChlb transformants was low compared to other photosynthetic organisms, Chl*b* played significant roles in photosynthesis. The importance of Chl*b* is not limited to its function as an accessory chlorophyll. Both Chl*b* and its biosynthetic enzyme CAO play important roles in the proper assembly of antennal complexes, in addition to the accessory functions of Chl*b* in LHC, contributing to photosynthetic efficiency and growth in plants and green algae, particularly under ML conditions [5, 6, 33]. Lack of Chl*b* or mutations in CAO result in not only defective photosynthesis but also poor growth-related phenotypes including leaf senescence and stress sensitivity [48]. It would be worthwhile to analyze these beneficial phenotypes in our CrCAO transformants.

A detailed assessment of the photosynthetic parameters and proteins in CrCAO transformants allowed us to elucidate the mechanisms of the beneficial effects of heterologous expression of CrCAO. We found that the transformants showed increased efficiency of PSII [Y(II)] and relative electron transfer rate (rETR), suggesting enhanced photosynthetic efficiency of PSII. We observed variable increases in the PSII antenna and core components, but not in those of PSI. In particular, Lhcb4 (or CP29, a minor monomeric antennal component) was greatly increased in transformants under ML, which is known to be regulated and stabilized by CAO in plants [5, 33]. Thylakoid membrane lipids including MGDG and DGDG were also increased in transformants under ML, consistent with improved photosynthesis. These galactolipids have also been associated with chloroplast and cell division in plants [49, 50]. In the case of D1 protein, we noted that the transformants cultivated under HL showed 3.4 and 6.3-fold increase in the amount of D1 while no significant changes were observed under ML. It has been reported that D1 shows rapid turnover under high light conditions, and more susceptible to photo-inhibition than other photosynthetic proteins [51, 52]. Hence, we assume that Chl*b* may have protected the D1 protein from degradation under HL, as similar functions have been reported for Chl*a* and Zn-pheophytin in plants [52]. However, there should be other unknown reasons for the D1-related functions of Chl*b*, since the dimeric D2 was not affected in our transformants. This requires more studies, since it is the first time to produce Chl*b* in *Nannochloropsis*.

In addition, NPQ was not affected by the expression of CrCAO, suggesting that the novel Chl*b* does not influence photoprotection. We also observed a moderate but significant decrease in antennal and core proteins, the significance of which is unknown.

Another important improvement in CrCAO transformants was increased biomass and lipid productivity. The improved photosynthetic efficiency apparently increased growth of transformants, resulting in increased biomass especially under ML conditions. This is expected to be effective for outdoor cultivation, as the intensity of the sun varies in time, season, region, and weather, where Chl*b* expressing mutants will have better chance for photosynthesis during any time with low intensity light. Indeed, there have been numerous researches on the importance of diffuse radiation compared to direct radiation on photosynthesis in plants [53, 54]. Even though we did not achieve increased lipid contents, this opens the opportunity of exploiting heterologous expression of CAO in industrial production of biofuels and biomaterials in *Nannochloropsis*. Improved photosynthesis is critical for successful production of materials from microalgae, because it provides fixed organic carbons and energy for all downstream metabolic processes [55, 56]. There have been numerous attempts to improve the metabolic processes per se, but with limited success [57–60]. Metabolic and/or genetic engineering should accompany photosynthetic improvements to support biosynthesis of materials. Our strategy of improving photosynthesis by heterologous production of Chl*b* provided an excellent proof of concept for industrial production of biomaterials. It should also be noted that our vector constructed for introducing proteins into the chloroplast in *Nannochloropsis* is a useful tool for chloroplast engineering in heterokonts.

## Conclusion

In the present study, we synthesized the chlorophyll *b* in *Nannochloropsis salina* for the first time by expressing CrCAO from *C. reinhardtii*. The catalytic region of CrCAO was sufficient to produce Chl*b* in *N. salina* with proper localization lead by signal and transit peptides retrieved from endogenous GCSL sequence. The Chl*b* producing transformants showed increased photosynthetic efficiency along with higher amount of LHCs related proteins. Moreover, the lipid productivity of the transformants were higher than WT by up to 43%, where the difference was more significant under lower light conditions. These findings revealed positive effects of Chl*b* on photosynthesis and growth in a Chl*b*-less organism, which can be readily employed in the production of biomass and biofuels.

## Methods

### Microalgal strains and maintenance conditions

The algal strains *Nannochloropsis salina* CCMP 1776 and *Chlamydomonas reinhardtii* CC-124 were obtained from NCMA (National Center for Marine Algae and Microbiota) and Chlamydomonas Resource Center, respectively. For maintenance of each strain, *N. salina* and *C. reinhardtii* were monthly subcultured on modified F2 N [15 g/L sea salt, 30 mg/L NaH_2_PO_4_·2H_2_O, 427.5 mg/L NaNO_3_, 10 mM Tris–HCl (pH 7.6), 5 mL/L trace metal mixture (3.15 g/L FeCl_3_·6H_2_O, 4.36 g/L Na_2_ EDTA·2H_2_O, 10 mg/L CoCl_2_·6H_2_O, 180 mg/L MnCl_2_·4H_2_O, 22 mg/L ZnSO_4_·7H_2_O, 9.8 mg/L CuSO_4_·5H_2_O, 6.3 mg/L Na_2_MoO_4_·2H_2_O), and 2.5 mL/L vitamin stock (1 mg/L vitamin B12, 1 mg/L Biotin, 200 mg/L thiamine∙HCl)] agar plates and TAP (2.42 g/L Tris, 0.375 g/L NH_4_Cl, 0.1 g/L MgSO_4_·7H_2_O, 0.05 g/L CaCl_2_·2H_2_O, 0.0108 g/L K_2_HPO_4_, 0.0054 g/L KH_2_PO_4_, 1 mL/L glacial acetic acid, and 1 mL/L Hutner’s trace elements (50 g/L Na_2_EDTA·2H_2_O, 22 g/L ZnSO_4_·7H_2_O, 11.4 g/L H_3_BO_3_, 5.06 g/L MnCl_2_·4H_2_O, 1.61 g/L CoCl_2_·6H_2_O, 1.57 g/L CuSO_4_·5H_2_O, 1.10 g/L (NH_4_)6Mo_7_O_24_·7H_2_O, and 4.99 g/L FeSO_4_·7H_2_O) agar plates at 25 °C under continuous illumination of 100 μmol/m^2^/s.

### Vector construction

The total RNA of *C. reinhardtii* was extracted at the mid-exponential phase from 200 mg cell by using an RNeasy Plant mini kit (Qiagen, USA), followed by additional treatment with a DNA-free DNase kit (AMBION, USA) to eliminate any DNA remaining in the product. The RNA was then used as a template to produce cDNA by reverse transcription with Superscript™ III Reverse Transcriptase (Invitrogen, USA) and an oligo (dT)_20_ primer (Invitrogen, USA). The coding sequence of *CrCAO* gene (GenBank accession: XM_001690123.1) was amplified through polymerase chain reaction from the cDNA with CA_1/CA_2 and CA_3/CA_4 primers (Additional file 2: Table S1).

The signal and transit sequences ligated at the N-terminus of the *CrCAO* were predicted by SignalP [61, 62] and ChloroP [63] from the GCSL gene of *N. salina*. These signal and transit sequences were artificially synthesized in the form of CA1 and BK1 primers, each harboring half of the full leader sequence. The backbone of pNsChlb was amplified from a pNssfCherry vector from our previous study [64] using primers BK1 and BK2 and it contained the TUB promoter/terminator set for the expression of *CrCAO* gene. The selection marker Sh*ble* was also harbored in the backbone sequence regulated by the UEP promoter/terminator set. The PCR products were assembled altogether into the pNsChlb vector by the Gibson assembly technique [65].

### Transformation through electroporation

For the transformation of *N. salina*, we performed electroporation based on a previously published report with slight modifications. Cells were grown for 4 days under continuous illumination (120 μmol/m^2^/s) to the mid-exponential phase for harvesting. The harvested cells were then concentrated to 5 × 10^9^ cells/mL after washing three times with 375 mM sorbitol to eliminate any salts left in the cell. For transformation with an ECM 850 square Wave Electroporation System (Bio-Rad, USA), 50 μL of concentrated cells was placed in 0.2-cm gap cuvettes (BTX, USA) with 2 μg of linearized vector DNA. The electroporation was conducted under the following conditions: 2400 V voltage, 100 μs pulse length, and 50 successive pulses at an interval of 500 ms. Electroporated cells were recovered in modified F2 N media for 15 h at 25 °C under a dark condition. Transformed cells were spread on F2 N agar plates containing 2.5 μg/mL Zeocin (Invitrogen, USA) for selection after 4 weeks.

### Genomic PCR and RESDA-PCR

The genomic DNAs of wild-type and pNsChlb transformants were extracted with Instagene Matrix (Bio-Rad, USA) by slightly modulating the manufacturer’s guide manual for bacteria. Cells picked from the agar plate were resuspended in 1 mL of autoclaved water and centrifuged at 13,000 rpm for 1 min. After removing the supernatant, 200 μL of Instagene matrix was added into the pellet and incubated at 56 °C for 10 min, followed by 8-min incubation at 99 °C. The supernatant of the mixture was then collected by centrifugation and was used as a template for genomic PCR and RESDA-PCR. For genomic PCR, S1/S2 and SR1/SR2 sets of primers were used to detect the shble and 18srDNA, respectively.

RESDA-PCR was performed following protocols described by Kang et al. [64] except for the primers. For the first amplification step, degenerate primer DegTaqI and RE1 were used, where Q0 and RE2 primers were used for the second amplification step.

### Western blot analysis

A Western blotting analysis was conducted to confirm successful expression of Chlorophyll *a* Oxygenase in transformants and to examine any changes in the level of LHC-relevant proteins during cultivation. 1.5 × 10^8^ cells were harvested on the 8th day from inoculation and were mixed thoroughly with 100 μL of 1.5× modified Laemmli buffer (pH 7.6, 62.5 mM Tris–HCl, 25% glycerol, 5% β-mercaptoethanol, 7% sodium dodecyl sulfate (SDS), and 0.02% bromophenol blue) [66]. The mixture was then incubated at 99 °C for 7 min followed by centrifugation at 13,000 rpm at 4 °C. After centrifugation, the supernatants were collected and subjected to electrophoresis using Mini-PROTEAN^®^ Tetra Vertical Eletrophoresis Cell for Mini Precast Gels (Bio-rad, USA) along with Any kD Mini-PROTEAN^®^ TGX Stain-Free Gels (Bio-rad, USA) in 1× TGX buffer. The resolved proteins were blotted onto polyvinylidene difluoride (PVDF) membranes using Trans-Blot^®^ Turbo™ System (Bio-rad, USA). After blocking with phosphate buffer saline (PBS) containing 5% skim milk and 0.1% Tween 20, the transferred membranes were immunoblotted with primary and secondary antibodies sequentially. For primary antibodies, anti-FLAG-tag antibody (Cell Signaling Technology, USA) was used at a 1:1000 dilution rate to detect Flag-tag proteins at the C-terminal of transformants and anti-ATPβ synthase antibody (Agrisera, Sweden) at a 1:5000 dilution rate as a loading control. Additionally, the following light-harvesting complex related proteins were used for this research; Lhcb1 AS01-004 at 1:4000, Lhcb2 AS01-003 at 1:10,000, Lhcb3 AS01-002 at 1:2000, Lhcb4 AS06-117 at 1:5000, PsaA AS06-176 at 1:1000, PsaL AS06-108 at 1:1000, PsbA AS05-084 at 1:10,000, PsbD AS06-146 at 1:5000, and Cyt f AS06-119 at 1:2000 (Agrisera, Sweden). For secondary antibody, anti-rabbit IgG-horseradish peroxidase (HRP) conjugated antibody (Cell Signaling Technology, USA) was used at a 1:1000 dilution for all types of primary antibodies. Protein bands were visualized with a ChemiDoc system (Bio-Rad, USA) after treating the membranes with enhanced chemiluminescence (ECL) reagents (Bio-Rad, USA).

### Chlorophyll extraction and HPLC analysis

We harvested *N. salina* cells on the 8th and 12th days during cultivation and freeze-dried them for the analyses of chlorophylls and lipids. For sample preparation, lyophilized cells each of 5 mg were prepared in 2-mL tubes (Bertin Technologies, USA) with 0.1-and 0.5-mm-diameter zirconia/silica beads. After adding 1.1 mL of HPLC-grade acetone (Sigma-Aldrich, USA) in the tubes, samples were subjected to bead beating at 6000 rpm for 40 s using a bead beater (Percellys 24, Bertin Technologies, USA). In total 10 times of bead beating was applied for each samples, and to avoid pigment degradation by heat, cells were cooled down in ice after each cycles. After filtering the supernatant with a 0.20-μm RC-membrane syringe filter (Sartorius Stedim Biotech, Germany), chlorophylls were analyzed by high performance liquid chromatography (HPLC) (Dionex Ultimate 3000, Thermo Scientific, USA) with a UV detector (Ultimate 3000 VWD Variable Wavelength Detector, Thermo Scientific, USA) and an Acclaim 120 C 18, 5 μm column (Thermo Scientific, USA). For the mobile phase, a (A) methanol-0.5 M ammonium acetate mixture (80:20, v/v) and a (B) methanol-acetone mixture (70:30, v/v) were flowed at a rate of 0.8 mL/min. The ratio of solvents (A) and (B) flowing into the column was gradually changed with the elapse of time.

### Photoautotrophic cultivation

Wild-type and NsChlb transformants were cultivated under high light (200 μmol/m^2^/s) and mid light (90 μmol/m^2^/s) in a F2 N medium at 25 °C with 120 rpm agitation. Each baffled flask contained 200 mL of cells, and a carbon source was directly supplied into the flasks in the form of 2% CO_2_ at 0.5vvm. The growth was determined by optical density, cell density, and dry cell weight (DCW). For each analysis, an automated cell counter (Cellometer^®^ Auto X4, Nexcelom, USA), UV–VIS spectrophotometer (UV-1800, Shimadzu, Japan), and GF/C filter paper (Whatman, USA) were used. The DCW was calculated by weighing the filter paper before and after filtering cells, which went through a delicate washing and drying procedure.

### Total lipid extraction and HPLC analysis

For total lipid extraction, 10 mL of chloroform–methanol mixture (2:1, v/v) was added to 20 mg of lyophilized cells and then sonicated for 1 h. To remove proteins and carbohydrates, 2.5 mL of deionized water was added and then vigorously mixed for 10 min, followed by centrifugation at 4000 rpm to separate the organic phase from the inorganic phase. We only collected the organic phase at the lower layer and filtered it through a 0.20-μm RC-membrane syringe filter (Satorius Stedim Biotech, Germany) to remove any impurities. After filtration, total lipids were analyzed by a high performance liquid chromatography (HPLC) (Agilent 1260, Agilent, USA) with an ELSD detector (Agilent 1260 ELSD, Agilent, USA) and a Chromolith Performance-Si (100 × 4.6 mm I.D) column (Merck Millipore, USA). A modified method for the gradient system was used based on previous reports [67].

### Photosynthetic activity measurement

The absorption spectra of wild-type and NsChlb transformantswere simply measured from 400 to 800 nm using a UV–VIS spectrophotometer (UV-1800, Shimadzu, Japan).

The fluorescence yield and light-response curves were measured in vivo with Multi-color-PAM (Heinz-Walz, Germany) as previously described [68, 69]. Cultured cells were dark adapted for 20 min before analysis for all the reaction centers to be opened. The photosynthetic parameters including relative electron transport rate (rel. ETR), and effective quantum yield of PS II (Y(II)) were measured with increasing light intensities of 440 nm LED light with a step length of 3 min.

### Immunogold labeling and transmission electron microscopy

In order to examine the localization of the expressed CrCAO protein, immunogold labeling was used with transmission electron microscopy. Harvested cells of WT and NsChlb19 transformant were washed with PBS buffer before fixation in ice for 20 min with fixation agent (4% paraformaldehyde with 0.1% glutaraldehyde). After washing three times with PBS buffer, cells were harvested in PBS solution containing 1% gelatin. Then, washing step after centrifugation for 2 min at 3000 rpm was repeated, increasing the content of gelatin in PBS from 2.5%, 7.5% and to 10%. For the steps with 2.5% gelatin and 7.5% gelatin, cells were incubated at 37 °C for 10 min. In the last step with 10% gelatin, cells were incubated in ice for 20 min. The samples were then sliced into 0.5–1 mm, which were supplemented with 2.3 M sucrose at 4 °C for overnight. After slicing the sample into sections, primary antibody of anti-FLAG-tag antibody (Cell Signaling Technology, USA) was used, followed by secondary gold-labeled anti-rabbit IgG antibody (Abcam, UK). Transmission microscopy image was taken with Tecnai G^2^ spirit TWIN transmission microscope (FEI, USA).

## Additional files

**Additional file 1: Figure S1.** Sequences of signal and transit peptides from GCSL in *N. salina* and coding sequence of CrCAO.

**Additional file 2: Table S1.** Primers used in this study.

**Additional file 3: Figure S2.** The prediction of leader sequence and localization by computational methods.

**Additional file 4: Figure S3.** RESDA PCR of WT and NsChlb transformants using DegTaqI as a degenerate primer.

**Additional file 5: Figure S4.** Localization of CrCAO in a transformant (NsChlb19) examined with immuno-gold labelling and transmission electron microscopy (TEM).

## Data Availability

All data generated or analyzed during this study are included in this published article.

## Abbreviations

DCW: dry cell weight
FAME: fatty acid methyl ester
Chl: chlorophyll
CAO: chlorophyllide *a* oxygenase
cER: chloroplast endoplasmic reticulum
LHCs: light-harvesting complexes
GCSL: Glycine Cleavage System L protein
cTP: chloroplast transit peptide
ML: medium light
HL: high light
MAG: monoacylglycerol
MGDG: monogalactosyldiacylglycerol
DGDG: digalactosyldiacylglycerol
rETR: relative electron-transfer rate
NPQ: non-photochemical quenching
TAG: triacylglycerol
