# Supplementary material for: Heterologous synthesis of chlorophyll b in Nannochloropsis salina enhances growth and lipid production by increasing photosynthetic efficiency
Source: Biotechnol Biofuels. 2019 May 14;12:122. doi: 10.1186/s13068-019-1462-3 (PMC6515666; doi:10.1186/s13068-019-1462-3)
Supplement: Supplementary file 1 — Additional file 1: Figure S1. Sequences of signal and transit peptides from GCSL in N. salina and coding sequence of CrCAO. [file 13068_2019_1462_MOESM1_ESM.docx]

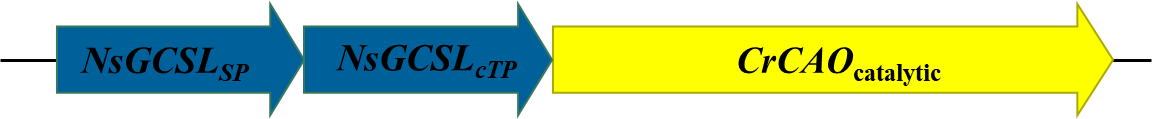


**> GCSL signal peptide in *N. salina***

ATGCGGTTGTCGTCGACCTGTCTTTTTCTGGCCATCACGGGGGCTACGGCT

**> GCSL chloroplast transit peptide in *N. salina***

TTCTTTTGCCCCAAACCCATGCCCCACCTGATGTCAGCGACTAGAGGCTTCGTCCCTGCGCATGTCGCGACGGCA

**>CrCAO catalytic domain from *C. reinhardtii***

ATGGAGATTGAGGAGGGCCTGCGCAACTTCTGGTACCCCGCTGAGTTCTCAGCGCGCTTGCCGAAGGACACGCTGGTGCCCTTTGAGCTGTTTGGCGAGCCGTGGGTGATGTTCCGTGATGAGAAGGGGCAGCCCTCCTGCATCCGCGACGAGTGCGCACACCGCGGCTGCCCGCTCAGCCTGGGCAAGGTGGTGGAGGGACAGGTCATGTGCCCCTACCACGGCTGGGAGTTCAACGGCGACGGCGCCTGCACCAAGATGCCCTCCACGCCCTTCTGCCGCAATGTGGGCGTTGCCGCGCTGCCTTGCGCGGAGAAGGATGGCTTCATCTGGGTCTGGCCCGGCGACGGCCTGCCAGCGGAGACGCTGCCGGACTTCGCCCAGCCGCCAGAGGGCTTTCTGATCCACGCGGAGATCATGGTGGATGTGCCTGTGGAGCACGGCCTGCTGATTGAGAACCTGCTGGACCTGGCGCACGCGCCGTTCACGCACACCAGCACCTTCGCGCGCGGCTGGCCTGTGCCCGACTTCGTCAAGTTCCATGCCAACAAGGCGCTCTCGGGCTTCTGGGACCCCTACCCCATCGACATGGCCTTCCAGCCGCCCTGCATGACGCTGTCCACCATCGGCCTGGCGCAACCCGGCAAGATTATGCGCGGCGTGACCGCCAGCCAGTGCAAGAACCACCTGCACCAGCTGCACGTGTGCATGCCCTCCAAGAAGGGCCACACGCGGCTGCTGTACCGCATGAGCCTGGACTTCCTGCCCTGGATGCGCCACGTGCCCTTCATCGACCGCATCTGGAAGCAGGTGGCGGCGCAGGTGCTGGGCGAGGACCTGGTGCTGGTGCTGGGCCAGCAGGACCGCATGCTGCGCGGCGGCAGCAACTGGTCCAACCCCGCGCCCTACGACAAGCTGGCGGTGCGCTACCGCCGCTGGCGCAACGGCGTAAACGCCGAGGTCGCACGCGTGCGCGCCGGCGAGCCACCGTCCAACCCCGTGGCAATGAGCGCGGGCGAGATGTTCTCGGTGGACGAGGATGACATGGACAAC

**Figure S1.** Sequences of signal and transit peptides from GCSL in *N. salina* and coding sequence of CrCAO.
