# Supplementary material for: Heterologous synthesis of chlorophyll b in Nannochloropsis salina enhances growth and lipid production by increasing photosynthetic efficiency
Source: Biotechnol Biofuels. 2019 May 14;12:122. doi: 10.1186/s13068-019-1462-3 (PMC6515666; doi:10.1186/s13068-019-1462-3)
Supplement: Supplementary file 2 — Additional file 2: Table S1. Primers used in this study. [file 13068_2019_1462_MOESM2_ESM.docx]

**Table S1.** Primers used in this study

| **Primer** | **Direction** | **Sequence (5' - 3')** |
| --- | --- | --- |
| **GC1** | **Fwd** | **ATGCGGTTGTCGTCGACCTG** |
| **GC2** | **Rev** | **TTAATGAGCGGTGCGGCCGA** |
| **S1** | **Fwd** | **AAGTTGACCAGTGCCGTTCCGGTG** |
| **S2** | **Rev** | **CTCGGTCACGAAGTGCACGCAGTT** |
| **SR1** | **Fwd** | **GTCAGAGGTGAAATTCTTGG** |
| **SR2** | **Rev** | **AACTAAGAACGGCATGCAC** |
| **CA1** | **Fwd** | **CAAGAAGCTGTCTTTTTGTGAAGCATGCGGTTGTCGTCGACCTGTCTTTTTCTGGCCATCACGGGGGCTACGGCTTTCTTTTGCCCCAAACCCATGCCCCACCTGATGTCAGCGACTAGAGGCTTCGTCCCTGCGCATGTCGCGACGGCA** |
| **CA2** | **Rev** | **AAAAGTGGTCGGACAAAAGGAGTTTCACTTGTCGTCGTCGTCCTTGTAGTCGTTGTCCATGTCATCCTCGTCCAC** |
| **BK1** | **Fwd** | **GACTACAAGGACGACGACGACAAGTGAAACTCCTTTTGTCCGACCACTTTTACAATC** |
| **BK2** | **Rev** | **TGCCGTCGCGACATGCGCAGGGACGAAGCCTCTAGTCGCTGACATCAGGTGGGGCATGGGTTTGGGGCAAAAGAAAGCCGTAGCCCCCGTGATGGCCAGAAAAAGACAGGTCGACGACAACCGCATGCTTCACAAAAAGACAGCTTCTTGATATTGACC** |
