# Supplementary material for: Heterologous synthesis of chlorophyll b in Nannochloropsis salina enhances growth and lipid production by increasing photosynthetic efficiency
Source: Biotechnol Biofuels. 2019 May 14;12:122. doi: 10.1186/s13068-019-1462-3 (PMC6515666; doi:10.1186/s13068-019-1462-3)
Supplement: Supplementary file 3 — Additional file 3: Figure S2. The prediction of leader sequence and localization by computational methods. [file 13068_2019_1462_MOESM3_ESM.docx]

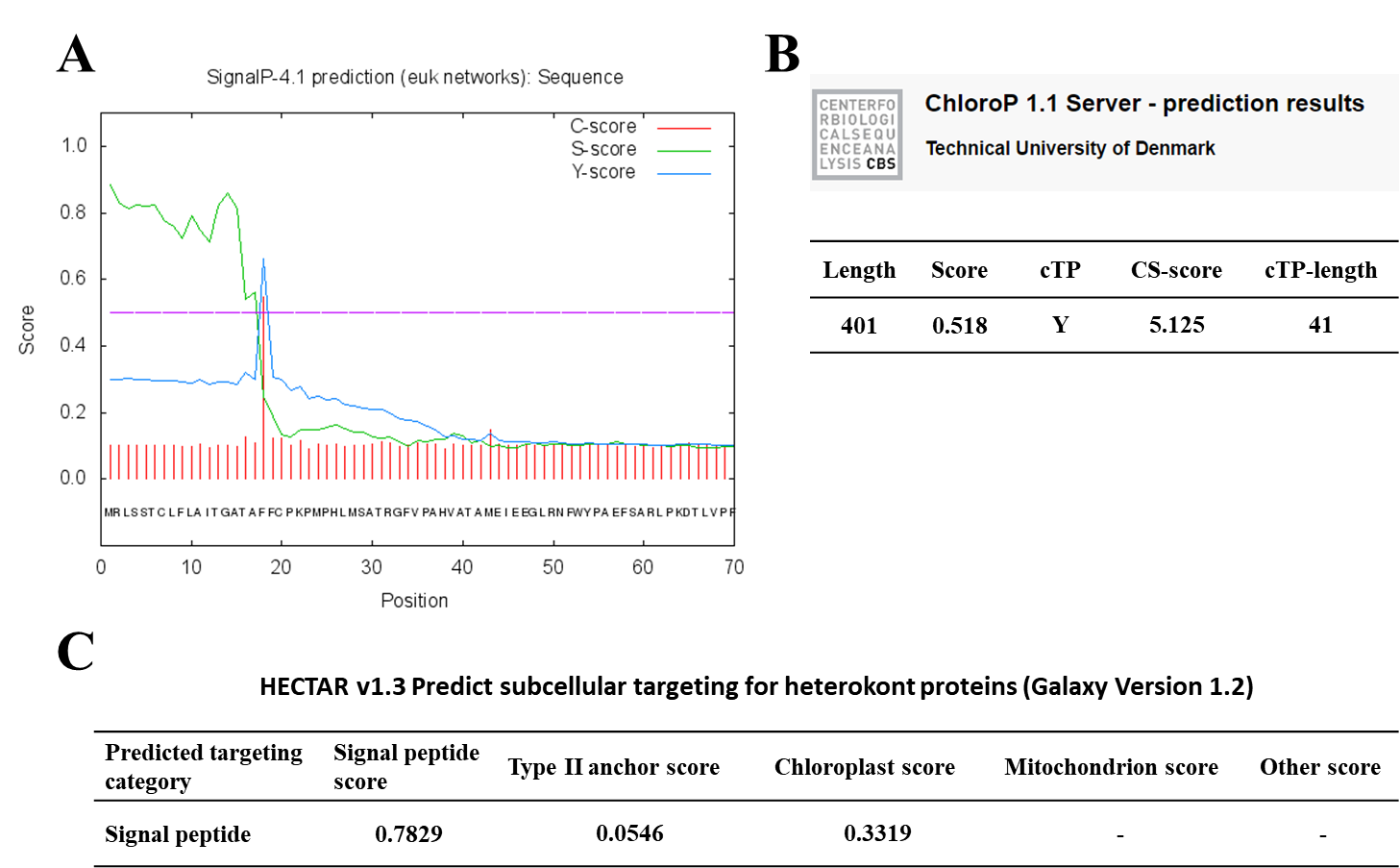


**Figure S2.** The prediction of leader sequence and localization by computational method. The signal peptide and transit peptide in chloroplast GCSL in *N. salina* were predicted by SignalP 4.1 **(A)** and ChloroP1.1 **(B)**, respectively. Localization of the chimeric CrCAO protein linked to the signal and transit peptide of *N. salina* was predicted to be localized in the chloroplast by HECTAR v1.3 **(C)**.
