# Supplementary material for: Heterologous synthesis of chlorophyll b in Nannochloropsis salina enhances growth and lipid production by increasing photosynthetic efficiency
Source: Biotechnol Biofuels. 2019 May 14;12:122. doi: 10.1186/s13068-019-1462-3 (PMC6515666; doi:10.1186/s13068-019-1462-3)
Supplement: Supplementary file 4 — Additional file 4: Figure S3. RESDA PCR of WT and NsChlb transformants using DegTaqI as a degenerate primer. [file 13068_2019_1462_MOESM4_ESM.docx]

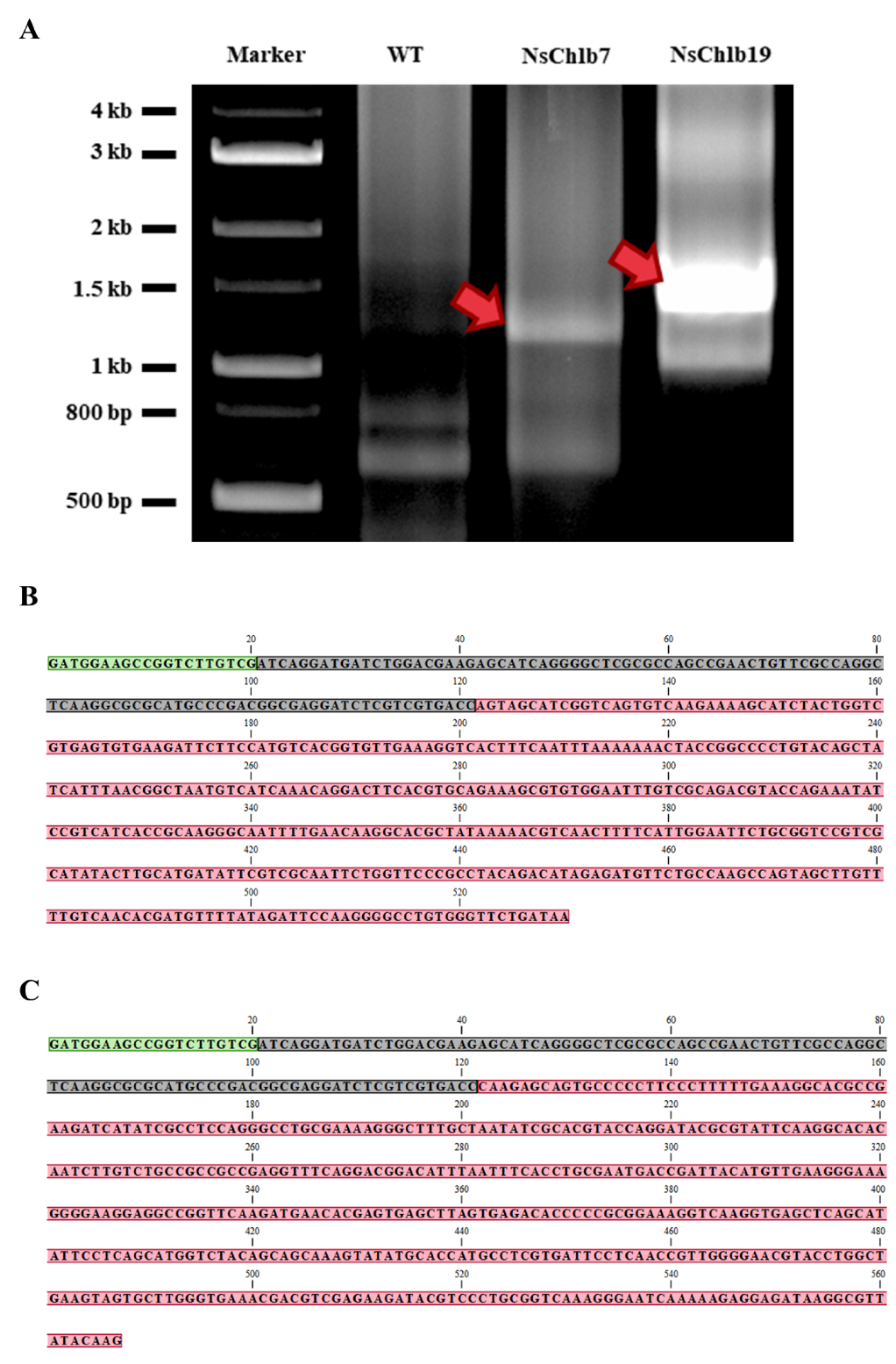


**Figure S3.** RESDA PCR of WT and NsChlb transformants using DegTaqI as a degenerate primer. Bands indicated with the red arrows were recovered and sequenced **(A)**. The sequence of the integration site in NsChlb7. The integration site was identified as a UTR region of a hypothetical protein (homologous to Naga_101464g1.1 in *N. gaditana* B-31) **(B)**. Integration sequence of NsChlb19. The integrated site is a intergenic region and is near protein phosphatase 2C (homologous to Naga_100033g27 in *N. gaditana* B-31). Integration sites were investigated from homology search of *N. gaditana* B-31 with *N. salina* using *Nannochloropsis* genome portal (<https://www.Nannochloropsis.org/>). The green, black, and red letters each represents sequences of the RESDA fwd2 primer, the vector, and genomic DNA. None of the transformants showed integration of vectors in coding regions.
