# Supplementary material for: Heterologous synthesis of chlorophyll b in Nannochloropsis salina enhances growth and lipid production by increasing photosynthetic efficiency
Source: Biotechnol Biofuels. 2019 May 14;12:122. doi: 10.1186/s13068-019-1462-3 (PMC6515666; doi:10.1186/s13068-019-1462-3)
Supplement: Supplementary file 5 — Additional file 5: Figure S4. Localization of CrCAO in a transformant (NsChlb19) examined with immuno-gold labelling and transmission electron microscopy (TEM). [file 13068_2019_1462_MOESM5_ESM.docx]

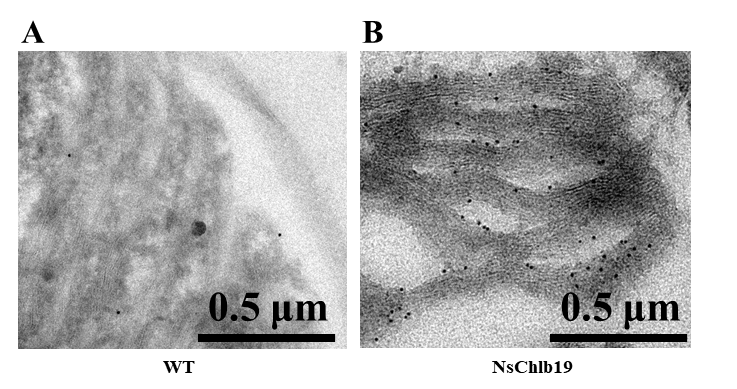


**Figure S4.** Localization of CAO in a transformant (NsChlb 19) examined with immuno-gold labelling and transmission electron microscopy (TEM). The localization of CAO is visualized as dots in the image, where no specific dots were found in the WT **(A)**. In contrast, numerous dots were detected in the NsChlb19 transformant mainly in the chloroplast along the thylakoid membrane **(B)**.
